# Supplementary material for: Synaptic vesicle traffic is supported by transient actin filaments and regulated by PKA and NO
Source: Nat Commun. 2020 Oct 21;11:5318. doi: 10.1038/s41467-020-19120-1 (PMC7578807; doi:10.1038/s41467-020-19120-1)
Supplement: Supplementary file 1 — Supplementary Information [file 41467_2020_19120_MOESM1_ESM.pdf]

Synaptic vesicle traffic is supported by transient actin filaments and regulated by PKA and NO

Authors

Nicolas Chenouard[1,2], Feng Xuan[1,3], Richard W. Tsien[1,4]

Affiliations

1. NYU Neuroscience Institute and Department of Neuroscience and Physiology, NYU Langone Medical Center, New York, NY 10016, USA.

2. Univ. Bordeaux, CNRS, Interdisciplinary Institute for Neuroscience, IINS, UMR 5297, F-33000 Bordeaux, France.

3. Current address: Interdepartmental Neuroscience Program, Northwestern University, Evanston, IL 60208, USA.

4. Center for Neural Science, New York University, New York, NY 10003, USA.

Corresponding author

R.W.T: Richard.Tsien@nyulangone.org

Supplementary Methods: image analysis

Extraction of the kymograph representation

In order to analyze spatio-temporal fluorescence variations along axons we used the method of kymograph (or kymogram) which consists in tracing the spatial location of an axon as a curved 2D line, and then extracting fluorescence through time along this line to obtain a 2D spatio-temporal representation of fluorescence. One dimension (vertical) is the locus along the axon, and time is the second one (horizontal). We used the plugin software ‘KymographTracker’ (plugin ID: ICY-K4O2C2) [1] for the ICY image analysis platform [2]. Briefly, for axon tracing we clicked on each axon extremities in a temporal projection of the image stack and the rest of the axon path was automatically traced by the software based on the local fluorescence intensity

using a Djikstra's (shortest path) algorithm and then smoothed based on a standard smoothing spline technique. At last, in each frame of the image sequence, fluorescence was extracted along the smooth 2D axonal path with a sampling step of 1 pixel to preserve the spatial scale. To enhance F-actin signals in Utr-CH:GFP kymographs, $\Delta F_{Utr\text{-}CH:GFP}$ measures were obtained by computing the differences between the raw Utr-CH:GFP fluorescence values and the pointwise averaged signals from -6 s to -1 s prior. We then applied a de-noising technique that smoothed images but preserved edge-like features [3].

Fluorescence auto-correlation analysis

For each image sequence, after kymograph extraction, we concatenated kymographs spatially as if axonal branches from the same field of view were a single structure and computed the Pearson linear correlation ρ between each pair of columns (time points) of the combined kymograph. The autocorrelation function value at time interval Δ*t* was computed as the median autocorrelation value of pairs of kymograph columns that are Δ*t* apart

$$r(\Delta t) = median_{K:kymographs}\rho(K(t), K(t + \Delta t))$$

The autocorrelation function $r(\Delta t)$ was fitted with a mono-exponential function $ae^{-\tau\Delta t} + b$, where τ was the decay rate (s^{-1}). τ characterized the overall fluorescence motility along the axonal branches: the larger, the less stable. In practice, the autocorrelation values are under-estimated because images are contaminated with a random (white) noise. In particular, $r(\Delta t \to 0) = k^2/(\sigma^2 + k^2) < 1$, where k^2 is the variance of the noiseless fluorescence signal and σ^2 is the variance of the noise. Importantly, white noise with constant power does not affect τ. Therefore, for display purpose only, we normalized autocorrelation functions $\hat{r}(\Delta t) = r\,(\Delta t)/r\,(\Delta t \to 0)$ so that image sequences with different noise levels are easier to visually compare. σ^2 was automatically estimated using the median absolute deviation measure and k^2 was computed as the average variance along kymograph columns minus σ^2.

Quantification of SV cluster dynamics

Starting from kymographs of fluorescence along axonal branches we traced high velocity events appearing as diagonal lines using ICY. As shown in Supplementary Fig. 4A, the width of segments corresponded to the duration of the event, height indicated distance traveled, and the slope was proportional to the speed. On top of extracting these three features characterizing individual trafficking events, we computed two global measures: the axonal *mobility* and *active transport (AT) rate*. For FM dyes and VAMP2:mCherry images they were computed as follows:

$$Mobility\ (s^{-1}) = \frac{Cumulated\ distance\ travelled\ by\ AT}{Total\ duration\ of observation \times length\ of\ monitored\ axonal\ segments}$$

$$AT\ rate\ (\mu m^{-1} s^{-1}) = \frac{Total\ number\ of\ AT\ events}{Total\ duration\ of\ observation \times length\ of\ monitored\ axonal\ segments}$$

where normalization was useful to compare image sequences with varying number of axonal branches and of density of synapses and vesicles. In contrast with FM dyes and VAMP2:mCherry, the QD-based staining is quantal so that it is appropriate to normalize measures by the staining rate (ie number of QDs). Because fluorescent traces are crisp, we were able to exhaustively trace paths for SV-QD clusters (Fig. S3A) and we computed the modified measures:

$$Mobility\ (\mu m s^{-1}) = \frac{Cumulated\ distance\ travelled\ by\ AT}{Total\ duration\ of\ observation \times number\ of\ QD\ clusters}$$

$$AT\ rate\ (s^{-1}) = \frac{Total\ number\ of\ AT\ events}{Total\ duration\ of\ observation \times number\ of\ QD\ clusters}$$

which account for the number of loaded QDs loaded in SVs and allowed us to fairly compare different field of views and coverslips with varying density of axons and synapses. AT segments where considered to be the ones with velocity >100 nm s^{-1}, in agreement with the single vesicle characterization of SV transport (Fig. 4).

Quantification of longitudinal actin polymerization

Actin polymerization was quantified in a similar fashion as for the analysis of FM 1-43 and VAMP2:mCherry puncta dynamics: kymographs were extracted based on the average Utr-CH:GFP fluorescence and polymerizing filaments were identified as diagonal lines (Supplementary Fig. 4B). Normalized measures of filament polymerization were computed as

$$Actin\ polymerization\ (s^{-1}) = \frac{Cumulated\ length\ of\ new\ actin\ filament\ polymerization}{Total\ duration\ of\ observation \times length\ of\ monitored\ axonal\ segments}$$

$$Filament\ count\ (\mu m^{-1} s^{-1}) = \frac{Number\ of\ new\ actin\ filaments}{Total\ duration\ of\ observation \times length\ of\ monitored\ axonal\ segments}$$

Single SV tracking and motion characterization

We first automatically detected the location of SV-QD using ICY (plugin ID ICY-R3M2Y2) [4] and refined the spatial localization by fitting a Gaussian approximation of the particle shape to the images (custom Matlab script). In order to quantify the localization accuracy, we adsorbed diluted QDs on a glass coverslip, applied the experimental imaging solution at 37C, and imaged immobilized QDs when using the same acquisition protocol as for trafficking experiments. Isolated QDs were then detected as above. Assuming detection errors are random and unbiased, the mean detected location over hundreds of frames should be an excellent approximation of the true location of the center of the immobile QD. For each frame we therefore computed the deviation from this pseudo-true location and estimated the localization error to be 30.2 nm on average (excluding frames in which QDs blinked off).

Trajectories of QDs were automatically built upon the detected positions using a computational technique that copes for blink off events and discards spurious detections (ICY plugin ICY-L5S9M5) [5]. We then systematically examined images to remove the few spurious trajectories which arose by chance from pixel noise. In each spatiotemporal trajectory, time points of AT initiation and termination were automatically detected using the trajectory analysis method described in [6]. Briefly, based on the vectors of Cartesian coordinates for each trajectory $[x(t)]_{t=0\ldots T-1}$ and $[y(t)]_{t=0\ldots T-1}$, we computed the 2D instantaneous displacements function: $d(t) = [x(t+1) - x(t), y(t+1) - y(t)]$ and a mathematical optimization method was used to approximate it by a 2D piecewise-constant function of time $v(t) = [v_x(t), v_y(t)]$ which corresponds to periods of constant velocity ($v(t) = v(t+1)$ for most t) and, doing so, cancels out fluctuations arising from random diffusion and localization inaccuracy. We then detected each period of constant velocity and tested them against a null model of random diffusion. Our model of AT is a sequence of steps with coherent direction and velocity, while random diffusion is a non-order set of displacements with arbitrary directions. In order to simulate diffusion with characteristics similar to the observed trajectory, we therefore repeatedly permuted observed displacements in a random order and changed their sign. We then compared the observed length of the cumulated displacement to the distribution of the simulated displacement lengths in order to compute the p-value of the putative AT period for the null model of diffusion. Only AT periods with $p<0.05$ were kept as non-diffusive.

In order to characterize the diffusive dynamics of SVs outside of AT events, we used the standard tool of mean squared displacements (MSDs). For a given time lag Δt and time segments devoid of AT (we split those containing AT in multiple parts), it consisted in first

computing the MSD length between all pairs of positions that are Δt apart in time

$$MSD(\Delta t)(\mu m^2) = \frac{1}{N}\sum_{t=0}^{T-\Delta t}\left(x(t+\Delta t)-x(t)\right)^2+\left(y(t+\Delta t)-y(t)\right)^2$$

with N the number of position pairs Δt apart in time. The shape of the MSD function was then analyzed as it characterized the mode of particle diffusion. For large Δt values it can be shown that the following model of MSD $MSD(\Delta t) \propto (\Delta t)^{\alpha}$or equivalently $logMSD(\Delta t) \approx \alpha log\Delta t + constant\ term$ is accurate for a variety of modes of diffusion [7]. α is a discriminant parameter characterizing diffusing type: α=1 for free normal diffusion, α>1 for super-diffusion (α=2 for directed motion such as AT), α<1 for anomalous sub-diffusive motion such as hindered, corralled or tethered diffusion [7]. We therefore computed the α value for each trajectory as the slope of a straight line fitted to the MSD log-log curve for Δt>1 s.

Syt1 cluster shape estimation

Fluorescent Syt1-IgG puncta were automatically detected in microscopy images and their center of mass (x_0, y_0) was identified with sub-pixel accuracy. We then fitted a two-dimensional Gaussian function

$$g(x,y) = Ae^{\frac{-(xcos\theta - ysin\theta - x_0)^2}{2L^2}}e^{\frac{-(xsin\theta + ycos\theta - x_0)^2}{2S^2}} + B$$

to the observed pixel intensity values around (x_0, y_0). Here, θ is a rotation angle with respect to the usual pixel Cartesian coordinates, L measures the longest puncta axis, and S is the shortest one.

Supplementary Table 1: Pharmacological compounds

Name	Provider	Catalog No.	Storage condition	Storage temperature
1,9-Dideoxyforskolin	Tocris Bioscience Bristol, UK	5034	5 mM in DMSO	-20C
8-Bromo-cGMP	Santa Cruz Biotechnology Dallas, TX	200316	50 mM in H_20	-20C
BHQ-3 amine	Biosearch Technologies Petaluma, CA	BHQ-3001-5	10 mM in DMSO	-20C
Colchicine	Sigma-Aldrich St Louis, MO	C9754	Desiccated	Room temperature
Cytochalasin D	Sigma-Aldrich St Louis, MO	22144-77-0	25 µg ml^{-1} in DMSO	-20C
DEA-NONOate	Cayman Chemical Ann Arbor, MI	372965-00-9	10 mM in degassed NaOH	-80C
Jasplakinolide	Tocris bioscience	2792	10 mM	-20C
Latrunculin A	Tocris bioscience	76343-93-6	100 µM in DMSO	-20C
ML 7	Tocris bioscience	4310	100 µM in DMSO	-20C
Nocodazole	Sigma-Aldrich	M1404	10 mM in DMSO	-20C
Okadaic Acid	Tocris Bioscience	78111-17-8	10 mM in DMSO	-20C
Y-27632 dihydrochloride	Tocris Bioscience	1254	10 mM in PBS	-20C

Supplementary Figures

Supplementary Figure 1

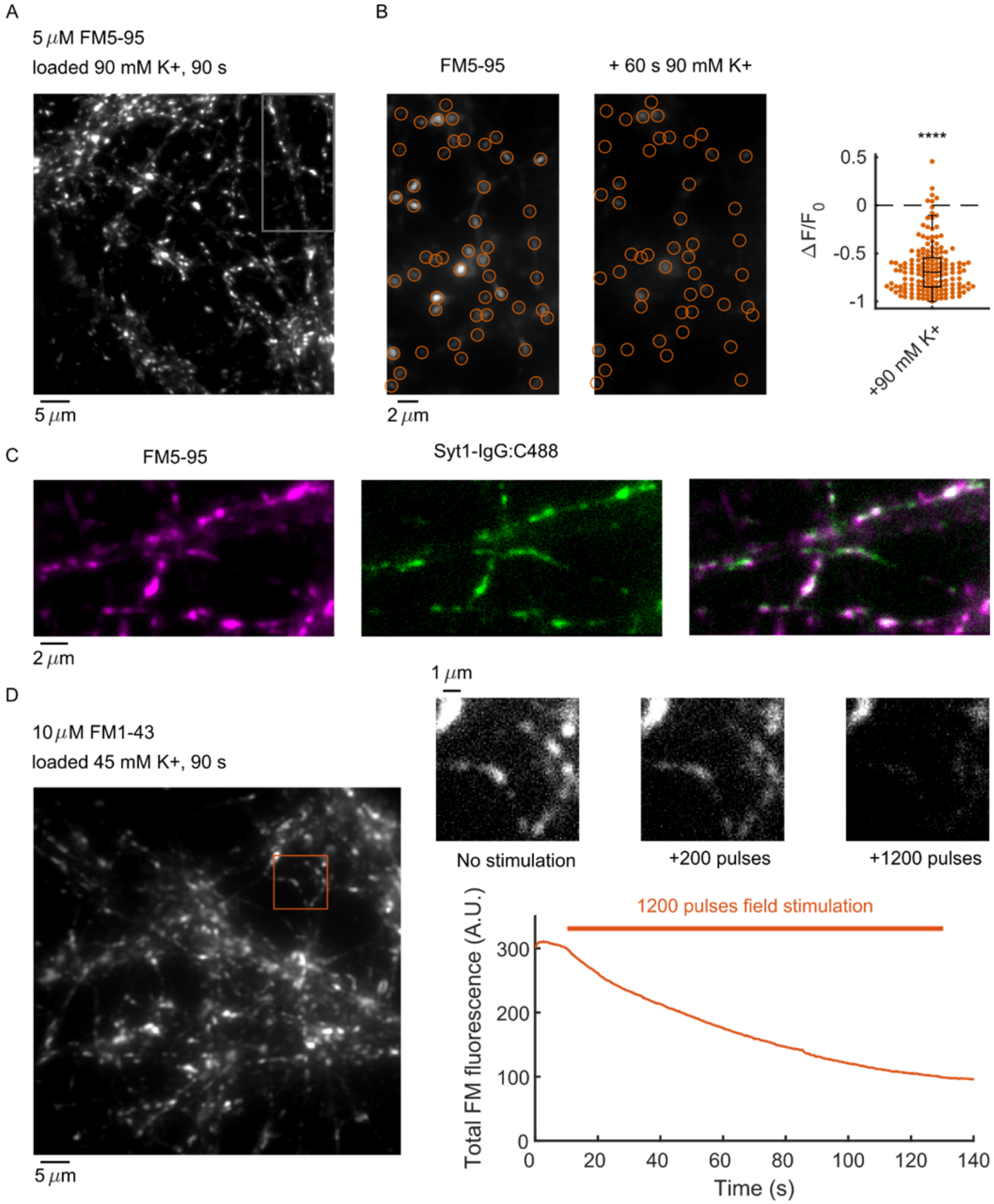

Supplementary Figure 1. FM dye molecules stained recycling SVs and were exocytosed upon both electrical and high K+ stimulations

A, C: When using a loading protocol based on high potassium stimulation, FM dye assumed a punctate distribution in cells and matched that of a fluorescent antibody against the lumenal domain of synaptotagmin 1, which was preloaded in synaptic vesicles (SVs), as expected from previous electron microscopy studies for specific SV targeting with FM dyes [8-11] and other vesicular probes [12]. C is a zoomed-in view of the gray box in A. B: A second high-potassium pulse triggered the exocytosis of FM dye molecules, attesting to the fusion-competence of SVs. Fluorescence in regions of interest automatically detected with ICY (red circles) strongly decreased after 90 s exposure to 90 mM K+ (median change -69.5%, $p<10^{-6}$, ones-sided sign (S) test, N=164 puncta from A). D: A 10 Hz train of electrical stimuli caused massive FM dye exocytosis. The onset of the dye loss matched the start of the stimulus train (red bar), hence confirming the electrically-triggered release of SVs.

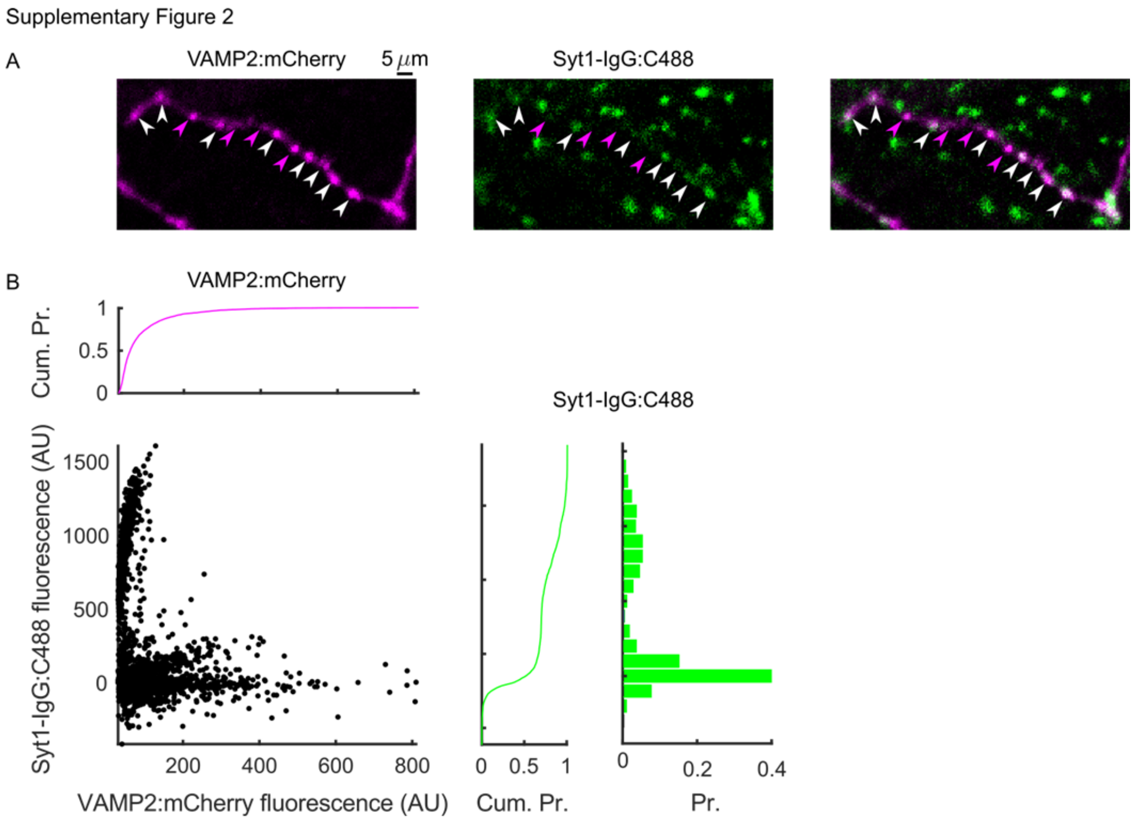

Supplementary Figure 2. Co-immunolabeling of VAMP2 and lumenal synaptotagmin 1

A: Live staining of SVs with a fluorescent antibody against the lumenal domain of synaptotagmin 1 (Syt1-IgG:C488) by spontaneous recycling (2-4 h) showed sites of colocalization with VAMP2-mCherry expression (white arrows) but also VAMP2-mCherry-positive puncta devoid of staining for recycling SVs (magenta). B: analysis of VAMP2:mCherry and Syt1-IgG:C488 fluorescence at detected IgG puncta showed two groups of puncta: high Syt1-IgG but low VAMP2:mCherry fluorescence (vertical cloud of points) and high VAMP2:mCherry but moderate Syt1-IgG:C488 fluorescence (right-leaning cloud). *N*=3274 puncta from 8 independent coverslips.

Supplementary Figure 3

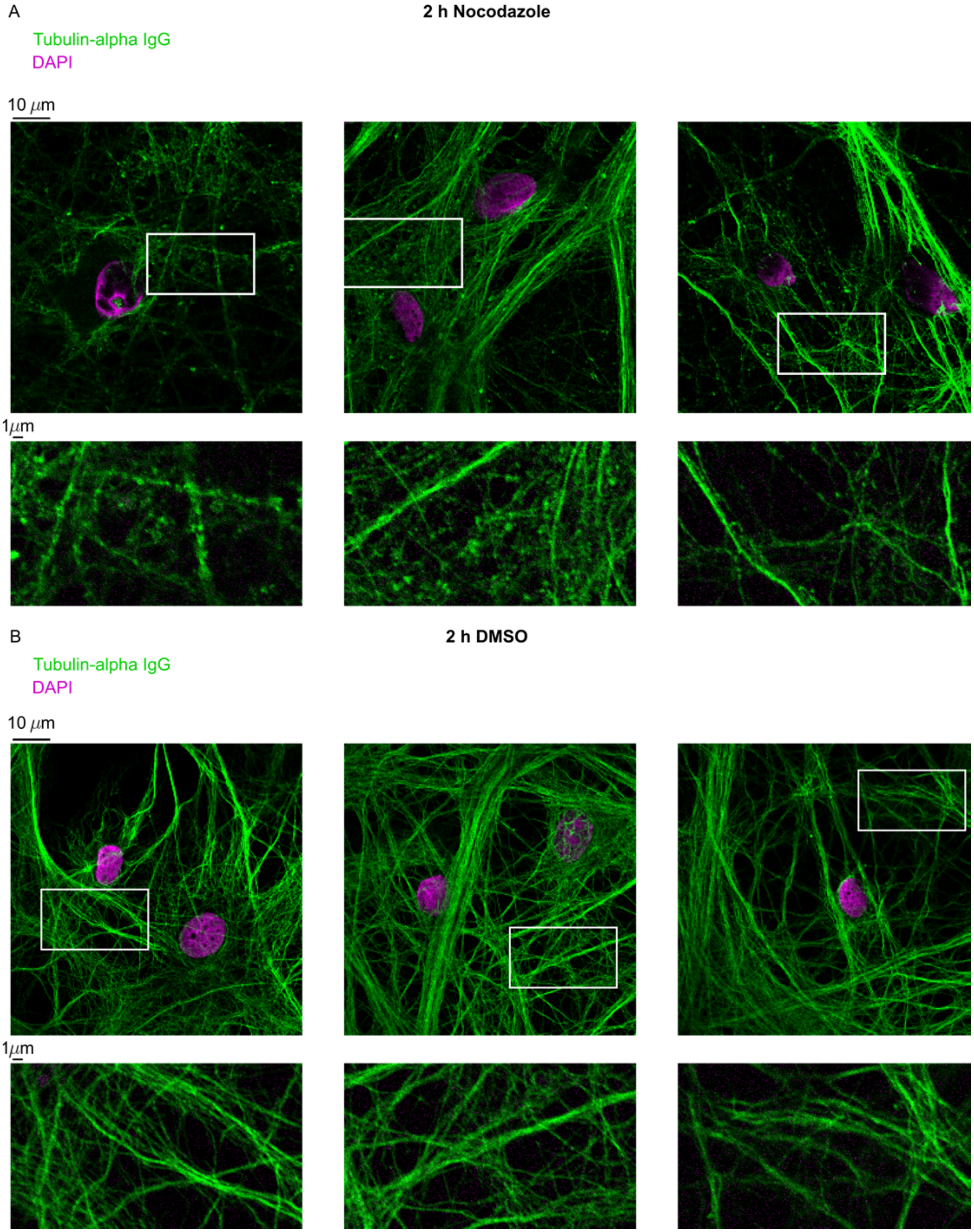

Supplementary Figure 3. Immunostaining for microtubules after nocodazole treatment

Cells were incubated for 2 h with either 10 µM nocodazole (A) or 1/1000 DMSO (B) in the culture medium at 37 C before being immunolabeled with an antibody against α-tubulin following the protocol described in Methods. Top row: exemplar full fields of view with confocal microscopy. Bottom row: insets corresponding to the white rectangles above. The contrast settings in the top row are consistent between conditions. They have been individually adjusted in bottom rows to facilitate the visualization of fine morphological details.

Supplementary Figure 4

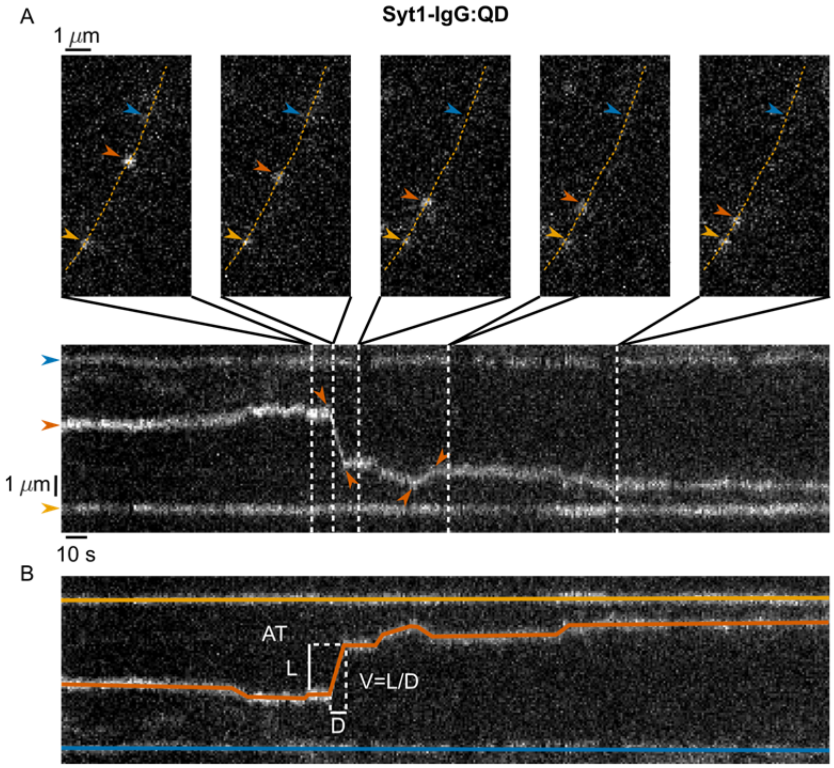

Supplementary Figure 4. Methods for characterizing unitary events of SV active transport along axons

A: Microscopy views of a short axonal segment labeled with Syt1-IgG:QDs for different time frames (top) and the corresponding kymograph representation (bottom). Three clusters of SVs labeled by QDs are indicated by colored arrowheads. The extraction path (axon) is outlined by the orange dotted curve. In the kymograph, red arrowheads indicate the starting and ending points of two active transport (AT) events. The semi-automated trajectory segmentation technique (Methods) yields the colored paths in (B). For one of the AT events we show the corresponding length (*L*, vertical size), duration (*D*, horizontal size) and the computation of the corresponding velocity (*V*).

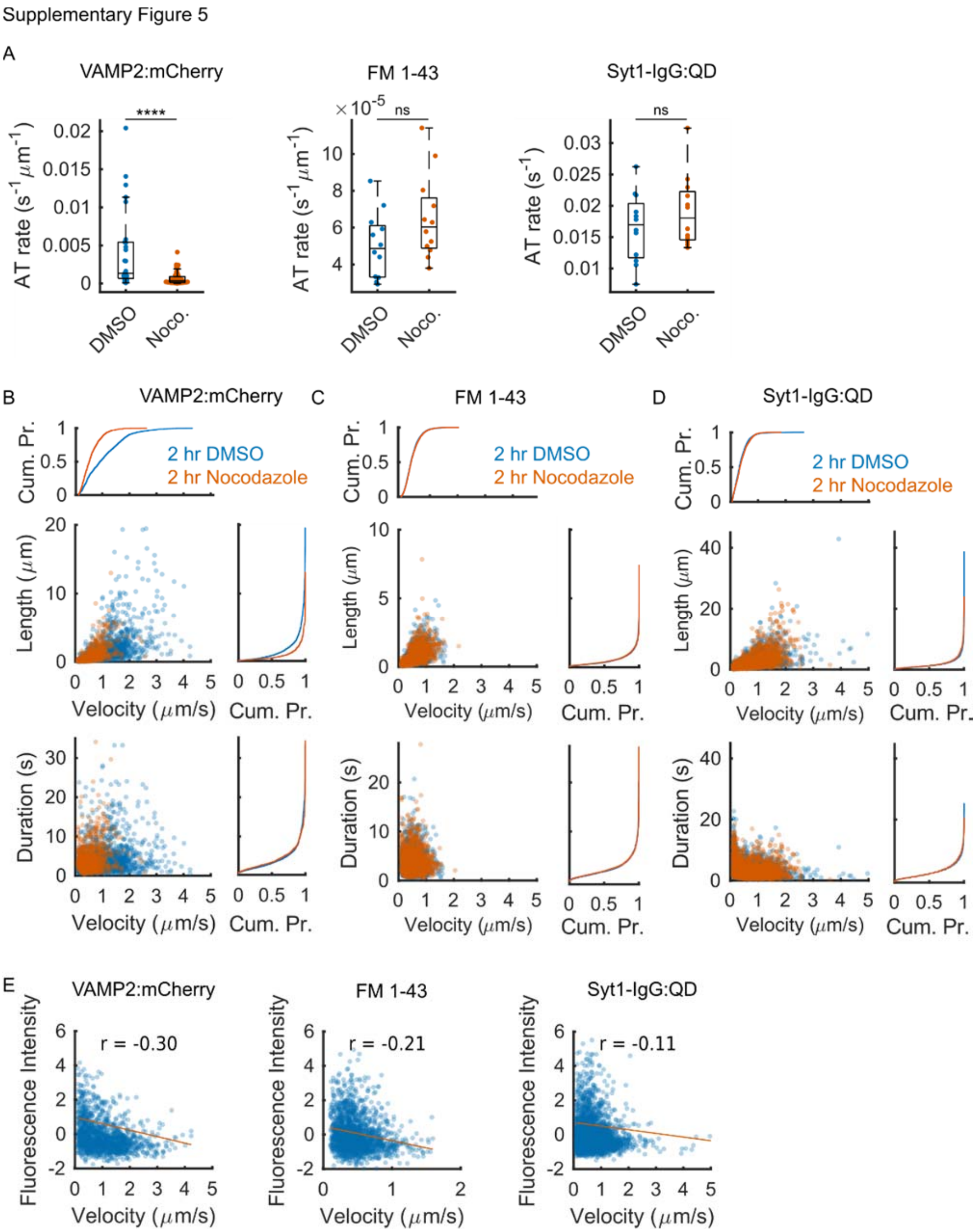

Supplementary Figure 5. Nocodazole decreased the unitary velocity and the rate of AT of VAMP2:mCherry cargos, but not of FM 1-43 and Syt1-IgG:QD

Kymographs of axonal traffic were extracted and analyzed to identify and characterize individual AT events after 2 h treatment with either 1/1000 DMSO or 10 μM nocodazole (Noco.) for the three fluorescent probes. Datasets for VAMP2:mCherry and Syt1-IgG:QD are the same as

main Fig. 1. A: The rate of AT events was diminished (median -74.5%) ($p<10^{-4}$, RS test; DMSO: *N*=12 movies from 8 coverslips; Noco.: *N*=12 movies from 4 coverslips) when VAMP2-mCherry was used. This was neither the case for SVs labeled with FM 1-43 (median +24.0%, $p>0.12$, rank-sum (RS) test; DMSO: *N*=12 movies from 5 coverslips; Noco.: *N*=12 movies from 3 coverslips) and Syt1-IgG:QDs (median +6.4%, $p>0.40$, RS test; DMSO: *N*=12 movies from 8 coverslips; Noco.: *N*=12 movies from 7 coverslips). B-E: B: Nocodazole had a significant effect on the unitary properties of AT events of VAMP2 clusters ($p<10^{-6}$, mANOVA including AT velocity, length and duration; DMSO: *N*=1468 events; Noco.: *N*=856 events). Velocity (median -44.5%, $p<10^{-6}$, RS test) and length (median -42.8%, $p<10^{-6}$, RS test) were the most affected (duration: median +9.3%, $p<5\times10^{-3}$, RS test). C-D: Nocodazole had no effect on the unitary properties of AT events of FM 1-43 clusters ($p=0.19$, mANOVA including AT velocity, length and duration; DMSO: *N*=1824; Noco.: N=2407 events) and of Syt1-IgG:QD-labeled cargos ($p=0.055$, mANOVA including AT velocity, length and duration; DMSO: *N*=2871 events; Noco.: *N*=2862 events). E: Pearson correlation between the velocity of cargos actively transported and their fluorescence intensity, a proxy for their physical size. VAMP2-mCherry: $r=-0.30$, ($p<10^{-6}$, *N*=1468 events). FM 1-43: r =-0.21 ($p<10^{-6}$, *N*=1824 events events). Syt1-IgG:QD: r =-0.11 ($p<10^{-6}$, *N*=2871 events events). Box plots: median, 25% and 75% percentiles (box) and extreme points (whiskers, excluding outliers).

Supplementary Figure 6

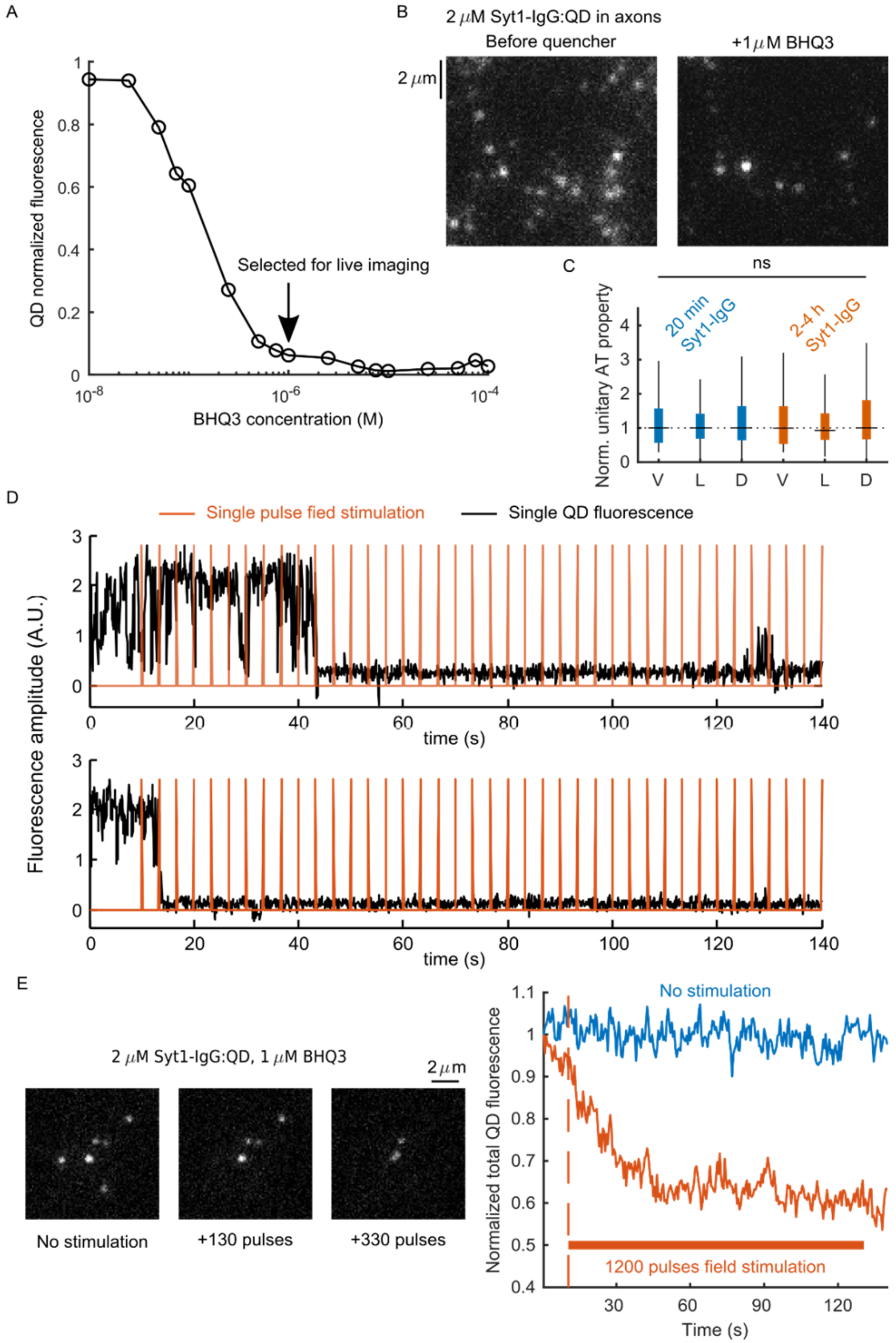

Supplementary Figure 6. Syt1-IgG:QDs were exocytosed upon electrical stimulation and the fluorescence of external QDs quenched

A: QD fluorescence quenching by BHQ3 was titrated in vitro by varying the concentration of BHQ3 in QD solutions in wells and using a fluorescence plate reader. B: In neurons, addition of 1 µM BHQ3 to the bath resulted in the disappearance of multiple QDs, confirming the potent quenching of the fluorescence of external QDs. C: The dynamics of SVs labeled with Syt1-IgG:QD with our standard protocol ('2-4 h Syt1-IgG', Methods) was to compared to SVs exposed for a shorter time to high concentration of IgGs ('20 min Syt1-IgG'). The latter protocol consisted in 1/ bathing cells in a Tyrode's solution with 25 mM K+ and containing synaptic AP5, NBQX, 4% BSA and the biotinylated anti-Syt1 antibody for only 20 min, 2/ washing the cells for 10 min with an antibody-free 4 mM K+ solution, 3/ applying a 45 mM K+ solution containing streptavidin-coated QDs and BSA for 90 s, 4/ extensively washing with a QD- and IgG-free solution. mANOVA including AT velocity (V), length (L) and duration (D): $p>0.3$ ('20 min', $N=284$ events from 4 coverslips; '2-4 h', $N=359$ events from 4 coverslips). D: When single electrical pulses were applied to cells by field stimulation, QD exocytosis appeared to be synchronized with the electrical stimulation. E: When a train of electrical stimuli (1200 stimuli, 10 Hz) was applied a significant number of QDs disappeared, hence confirming the fusion-competence of SVs labeled with Syt1-IgG:QDs. Fluorescence was stable in the absence of stimulation. We selected immobile puncta for the analysis (no stimulation $N=133$, stimulation $N=127$).

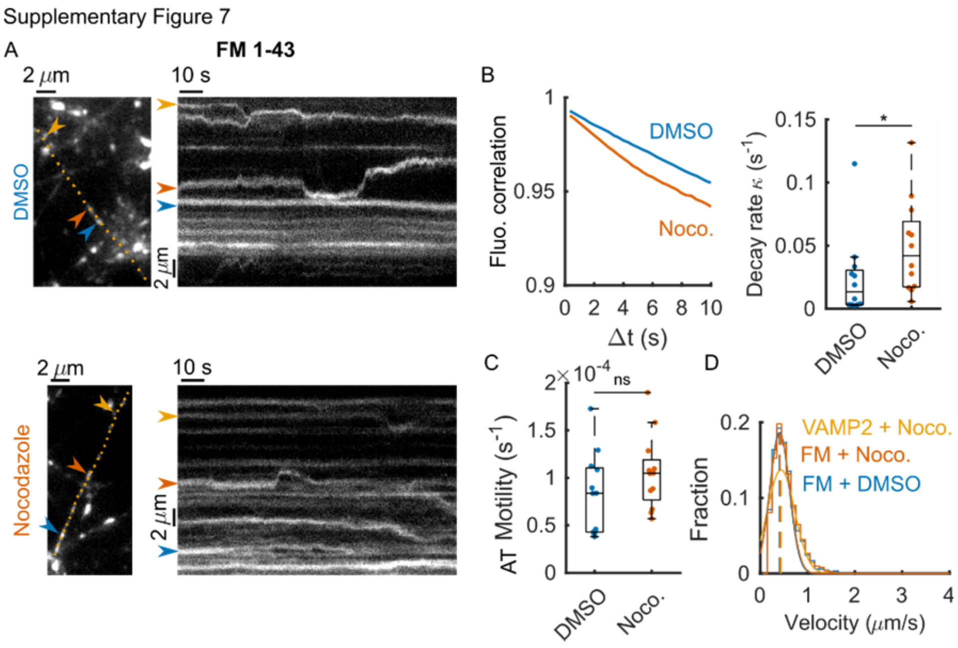

<u>Supplementary Figure 7. Microtubule disruption with nocodazole spared the transport of FM-labeled clusters</u>

FM 1-43 was endocytosed by neuronal cells after a standard hyper stimulation (45 mM K+, 90s) and imaged by time-lapse microscopy. A: Kymographs of FM-loaded SVs showed bright stationary clusters of fluorescence, likely corresponding to presynaptic terminals (blue arrowhead) along with mobile clusters that moved transiently (red and yellow arrowheads). Cultures were preincubated for 2 h with either 10 µM nocodazole (bottom) or 1/1000 DMSO (top). B: median autocorrelogram derived from kymographs (left) and corresponding individual exponential decay rates (right). The median auto-correlation decay rates were 0.0133 s^{-1} (DMSO, *N*=12 movies) and 0.042 s^{-1} (Noco., *N*=12 movies). *p*=0.04, RS test. C: the median AT-based motility was 1.05×10^{-4} (DMSO) and 0.84×10^{-4} (Noco.). *p*>0.19, RS test. D: The unitary velocity distributions were well-approximated by a single Gaussian function with mean 0.407±0.206 µm s^{-1} (DMSO, *N*=1824 events) and 0.414±0.210 µm s^{-1} (Noco., *N*=2407 events). Box plots: median, 25% and 75% percentiles (box) and extreme points (whiskers, excluding outliers). Data is from from 3 independent coverslips for the Noco. group and from 5 coverslips for DMSO treatment.

Supplementary Figure 8

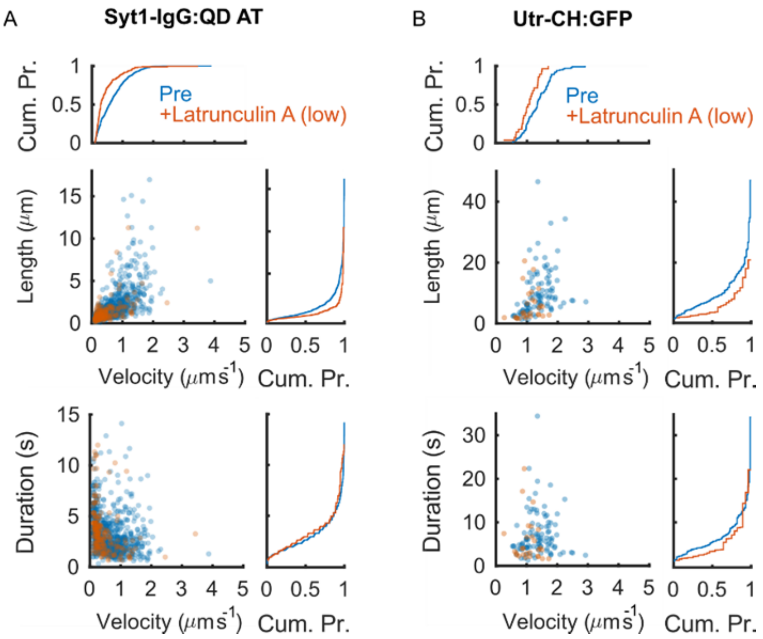

Supplementary Figure 8. Latrunculin A mainly affected the velocity of spared unitary SV AT events and actin filaments

The effect of a low dose of latrunculin A (0.1 μM, 3 min) (low) on the unitary properties of SV AT events (A) and on the unitary properties of actin filaments as visualized by Utr-CH:GFP [13] (B) were quantified. A paired experiment to minimize inter-coverslip variability was used. Raw data used for analysis was the same as in Fig. 2. A-C $p<10^{-6}$, mANOVA including AT velocity, length and duration (pre, N=772 events; latrunculin A, N=195 events). Length: $p<10^{-6}$, RS test. Velocity: $p<10^{-6}$, RS test. Duration: $p>0.10$, RS test. B: $p<10^{-2}$, mANOVA including AT velocity, length and duration (pre N=100 events; latrunculin A N=28 events). Length: $p<10^{-3}$, RS test. Velocity: $p=2\times10^{-3}$, RS test. Duration: $p<10^{-2}$, RS.

Supplementary Figure 9

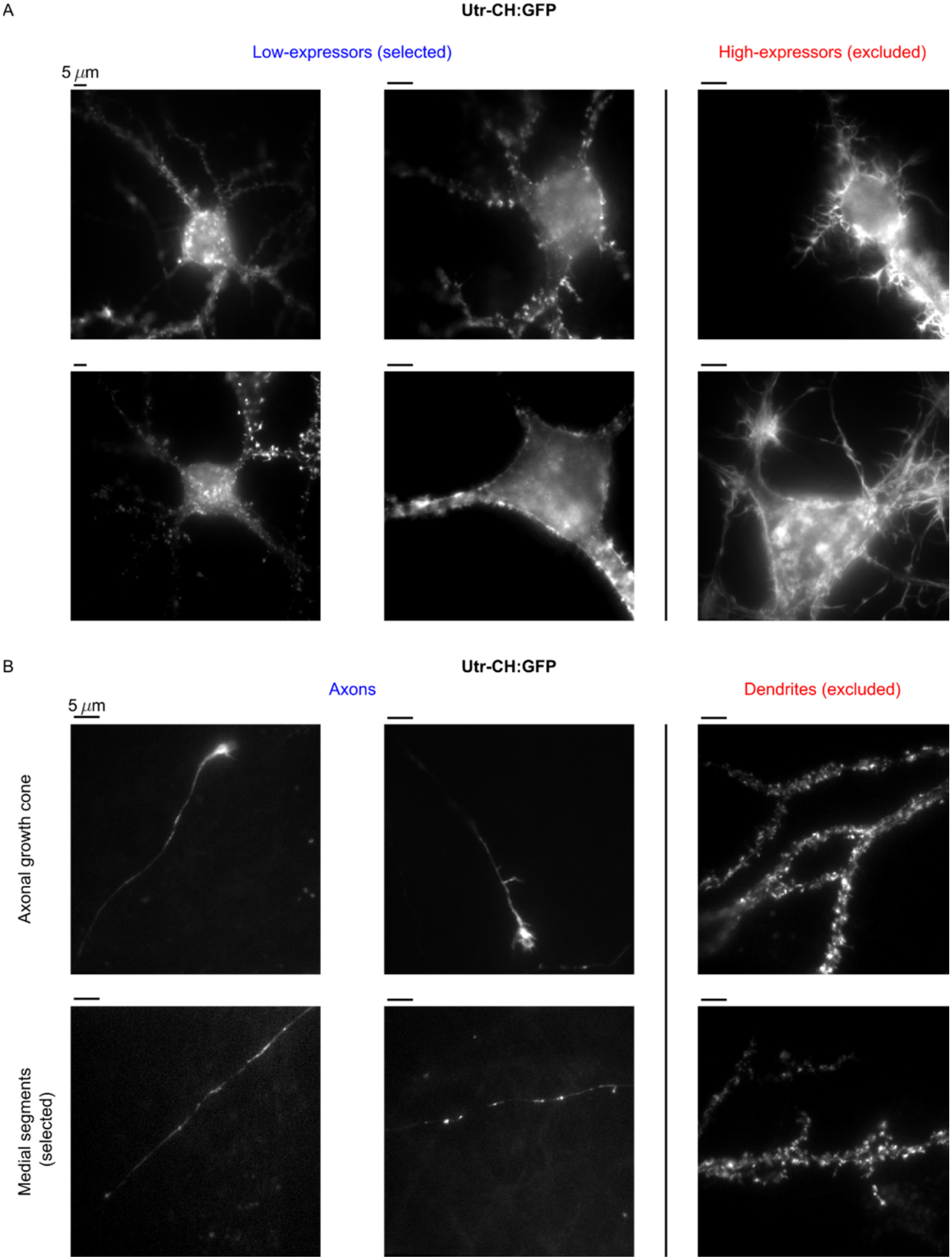

Supplementary Figure 9. Selection of axons expressing Utr-CH:GFP as a F-actin probe

As reported earlier, the level of Utr-CH:GFP expression in cultured hippocampal neurons can vary from one cell to another, with high amounts of Utr-CH:GFP impairing physiological actin dynamics [14]. Based on the level of fluorescence and the presence of morphological defects neurons were therefore systematically screened and segregated between high and low expressor cells (A) and only low expressors were selected for further experiments. B: axons were identified by finding stereotypical growth cones rich in actin (top left), and tracing back axons to their more medial part (bottom left). Medial parts assumed a smooth shape with bright clusters likely corresponding to synapses and axonal actin ‘hotspots’ [14]. Medial axonal segments could not be confused with dendrites which were thicker, branched, and contained bright spines sprouting out of the shaft (right). Image contrast was individually adjusted for clear visualization of fine morphological details.

Supplementary Figure 10

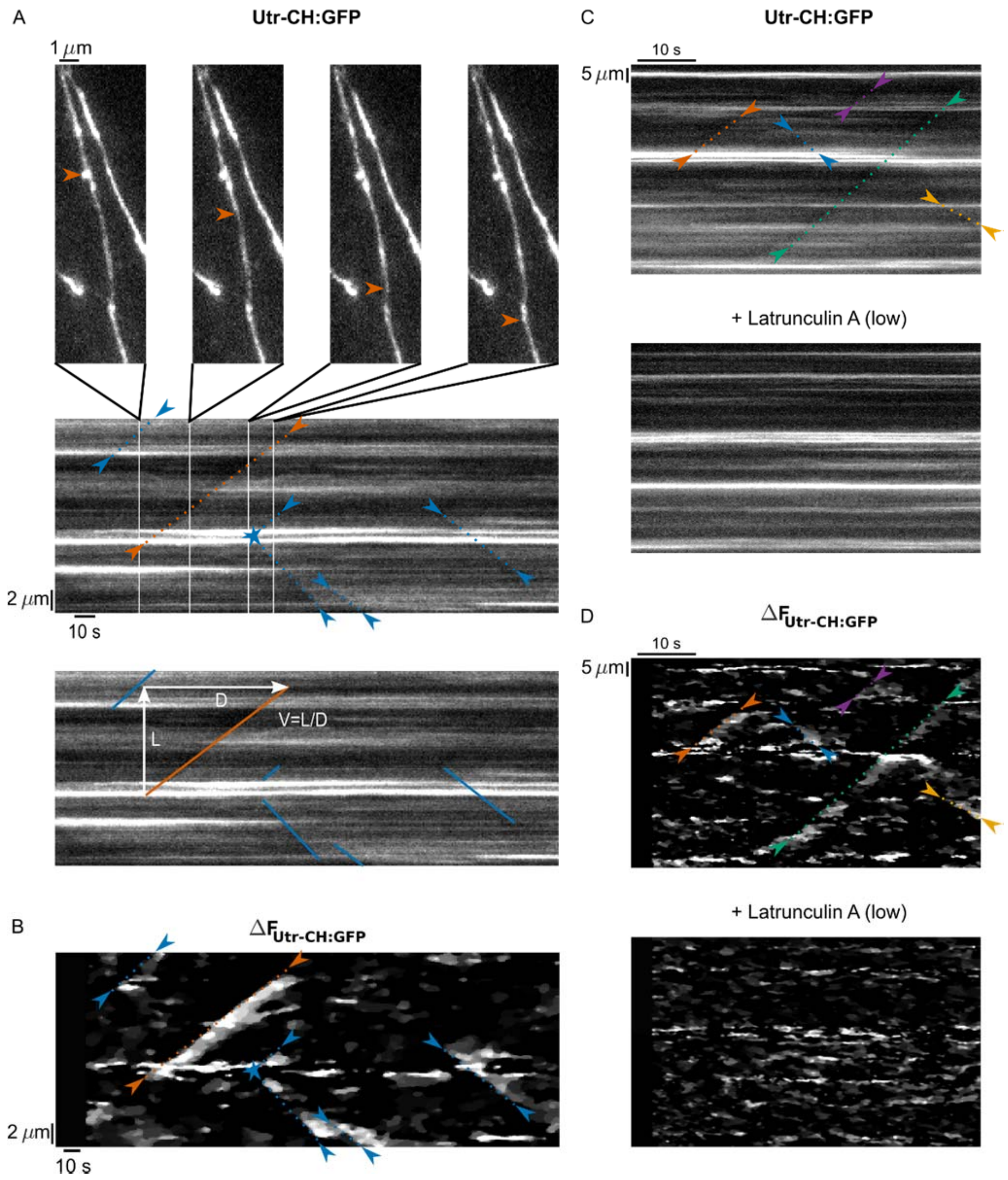

<u>Supplementary Figure 10. Quantification of longitudinal F-actin polymerization along axons and of the effect of latrunculin A (low)</u>

Longitudinal F-actin in axons was visualized by virtue of the expression of Utr-CH:GFP and the extraction of spatio-temporal kymographs, which enabled quantification of pharmacological effects. A: A filament tip (red arrow) was seen to emerge from an actin hotspot (top, left-most panel) and to quickly elongate along an axon (top-row panels). After kymograph extraction (middle panel), the filament was seen as a diagonal step of fluorescence increase (red line and

arrows). Multiple other polymerization events could be detected along the same axonal stretch (blue). Filament elongation length (*L*) and duration (*D*) were measured as the vertical and horizontal sizes of fluorescence steps and elongation velocity was computed as $V = L/D$ (bottom panel). B: $\Delta F_{Utr\text{-}CH:GFP}$, a filtered image that enhances step-like features in images was used to check the presence of the polymerization events identified in (A). $\Delta F_{Utr\text{-}CH:GFP}$ was computed as the smoothed, positive, difference between the UTR-CH:GFP fluorescence at the current time point minus the averaged signal from -6 s to -1 s prior (Methods). C: Exemplar UTR-CH:GFP signals before and after a treatment with a low dose (0.1 µM, 3 min) of latrunculin A. Same images as in main manuscript Figure 2C. D: $\Delta F_{Utr\text{-}CH:GFP}$ images corresponding to (C), showing the presence of F-actin polymerization events (diagonal edges, top panel) or their absence (bottom) prior to or after treatment with latrunculin A.

Supplementary Figure 11

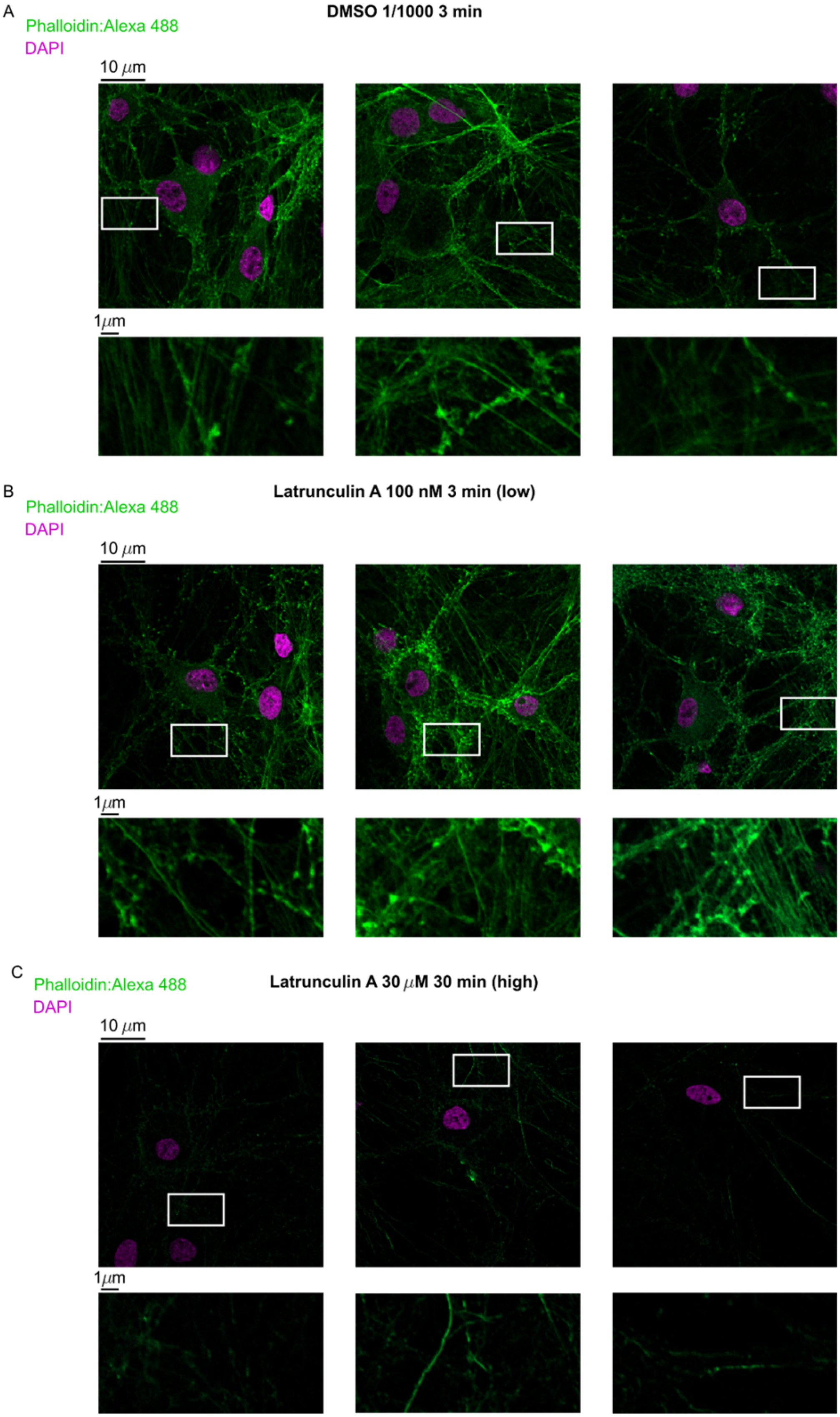

Supplementary Figure 11 (continued)

D

Cytochalasin D 25 μg ml^{-1} 30 min

Phalloidin:Alexa 488
DAPI

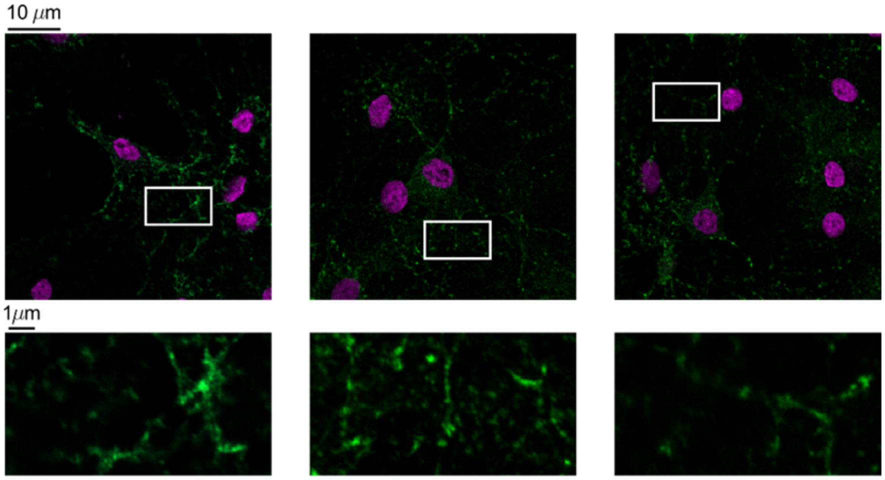

E

C3 transferase 15 μg ml^{-1} 2 h

Phalloidin:Alexa 488
DAPI

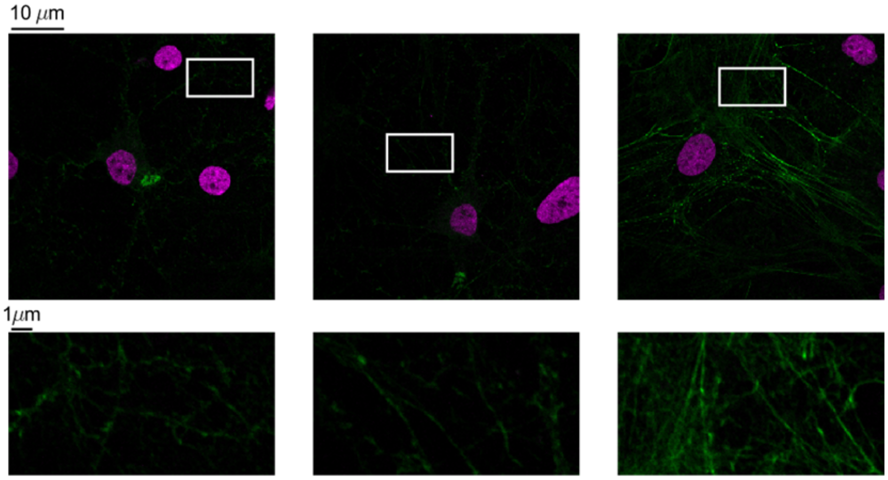

F

Y27632 10 μM 2 h

Phalloidin:Alexa 488
DAPI

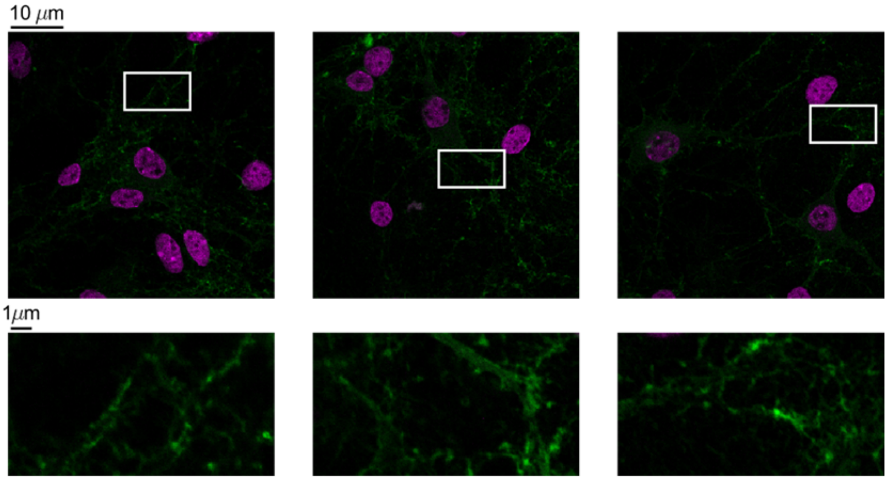

Supplementary Figure 11 (continued)

G

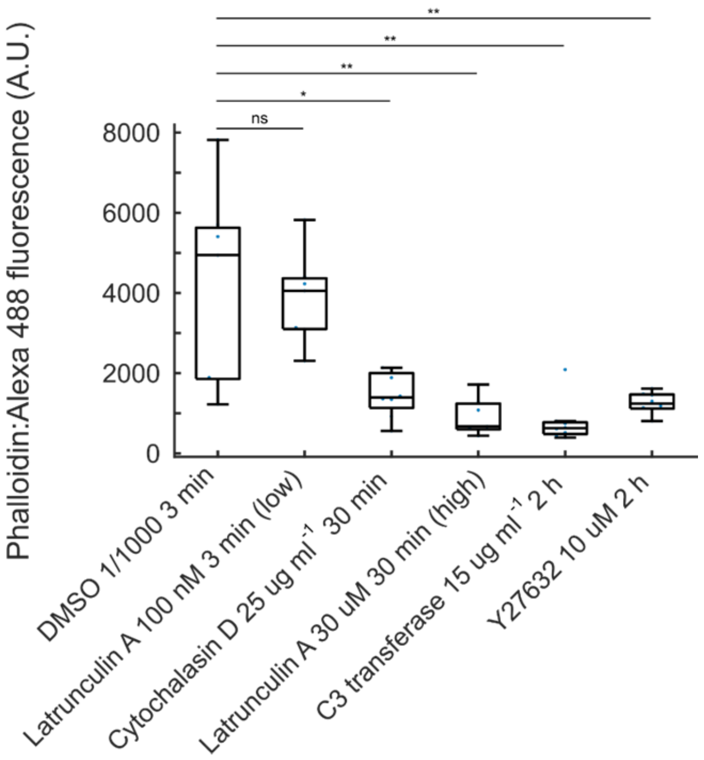

Supplementary Figure 11. Labeling of F-actin after differential application of actin-perturbing drugs

F-actin was labeled using a Phalloidin:Alexa 488 probe after G-actin extraction and fixation (Methods). Pharmacological protocols were similar to that of experiments in which SV-traffic and dynamic actin polymerization were monitored (Fig. 2 and 6). A-F: Top rows: exemplar full fields of view with confocal microscopy. Bottom rows: insets corresponding to the white rectangles above. The contrast settings in the top row are consistent between conditions. They have been individually adjusted in bottom rows to facilitate the visualization of fine morphological details. G: Total fluorescence was measured for each pharmacological treatment as a readout of total F-actin amount. Treatments were individually compared with the control condition (1/1000 DMSO, 3 min) (N=7). Latrunculin A (low): median fluorescence -18.0% (p=1, RS test) (N=7 images). Latrunculin A (high): median fluorescence -86.4% (p=0.002, RS test) (N=7 images). Cytochalasin D: median fluorescence -71.8% (p=0.05, RS test) (N=8 images). C3 transferase: median fluorescence -87.3% (p=0.002, RS test) (N=8). Y27632: median fluorescence -74.9% (p=0.004, RS test) (N=8 images). Box plots: median, 25% and 75% percentiles (box) and extreme points (whiskers, excluding outliers).

301

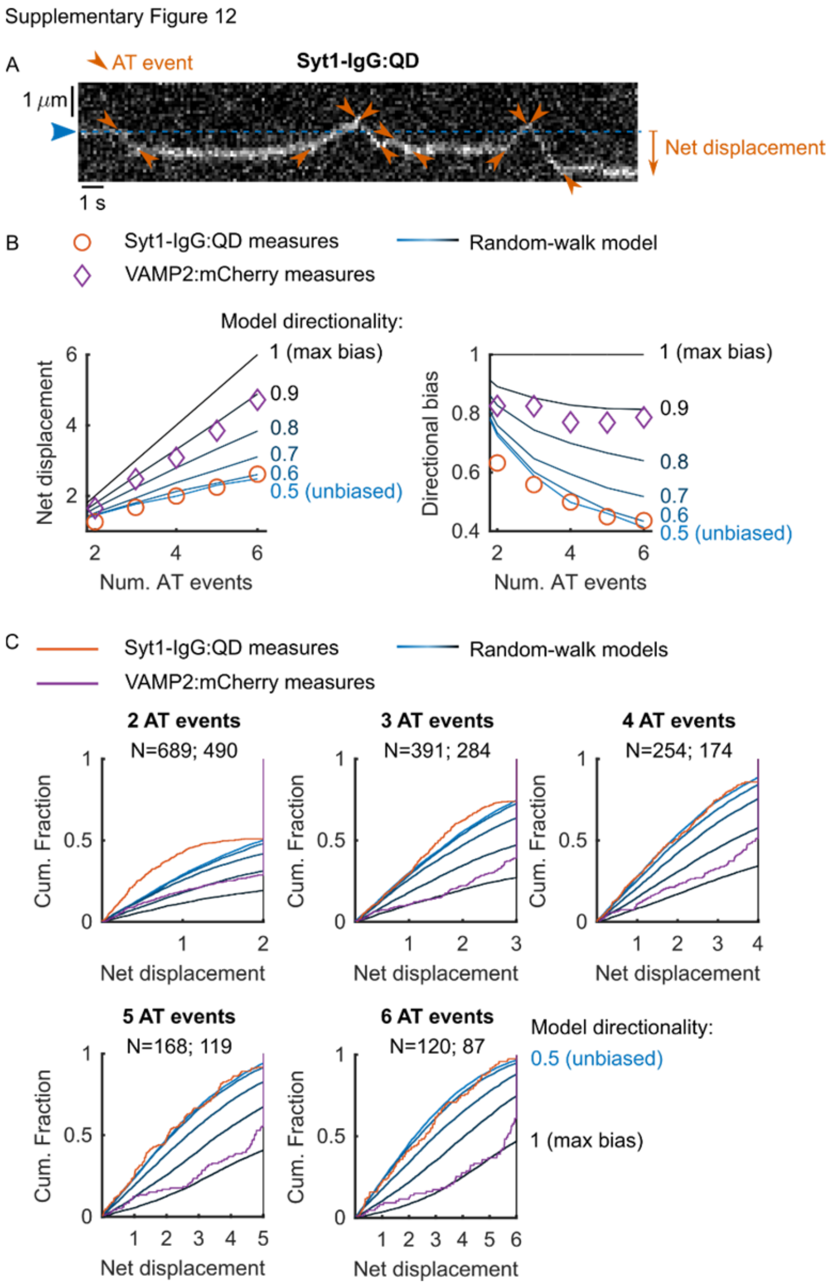

Supplementary Figure 12. Sequences of Syt1-IgG:QD active transport events are not directionally biased

A: For 327 Syt1-IgG:QD clusters, we measured the net AT-based displacement as the distance between the QD positions before and after a sequence of AT events. Net displacement measures were then rescaled individually by normalizing each AT length by the average AT distance. Therefore, after normalization, the maximum net distance for a sequence of *k* AT events was *k*. B: Net displacement values for different numbers of events were compared (red circles) to those obtained with unbiased random-walk models with different probabilities of

moving in the dominant direction (‘model directionality’, 1: fully directional, 0.5: unbiased) (simulated trajectory N=5000 for each directionality setting) (left, average). AT distance values for the random walk model were randomly drawn in a centered normal distribution and renormalized for unit average norm. The anticipated maximal net displacement in case of a maximally directional sequence of AT events is a unit-slope line (black line). To better measure the evolution of the directional bias with an increasing number of AT events we also renormalized net displacement values by the maximal directional displacement (right). The directional bias for Syt1-IgG:QD measures (N=262 SVs from 10 coverslips) did not appear to increase with the number of AT events and was indistinguishable from the unbiased random-walk model, in particular for long sequences of AT events which could more robustly highlight a possible directional bias. By contrast, the analysis of the AT of and 305 VAMP2:mCherry clusters (from 5 coverslips) provided evidence for strongly biased (~0.9) traffic (purple diamonds). C: The distributions of experimental net displacement measures were not significantly different from those of the unbiased random-walk for Syt1-IgG-containing clusters (6 AT events: p>0.34, RS test), but were for VAMP2:mCherry cargos (6 AT events: $p<10^{-6}$, RS test).

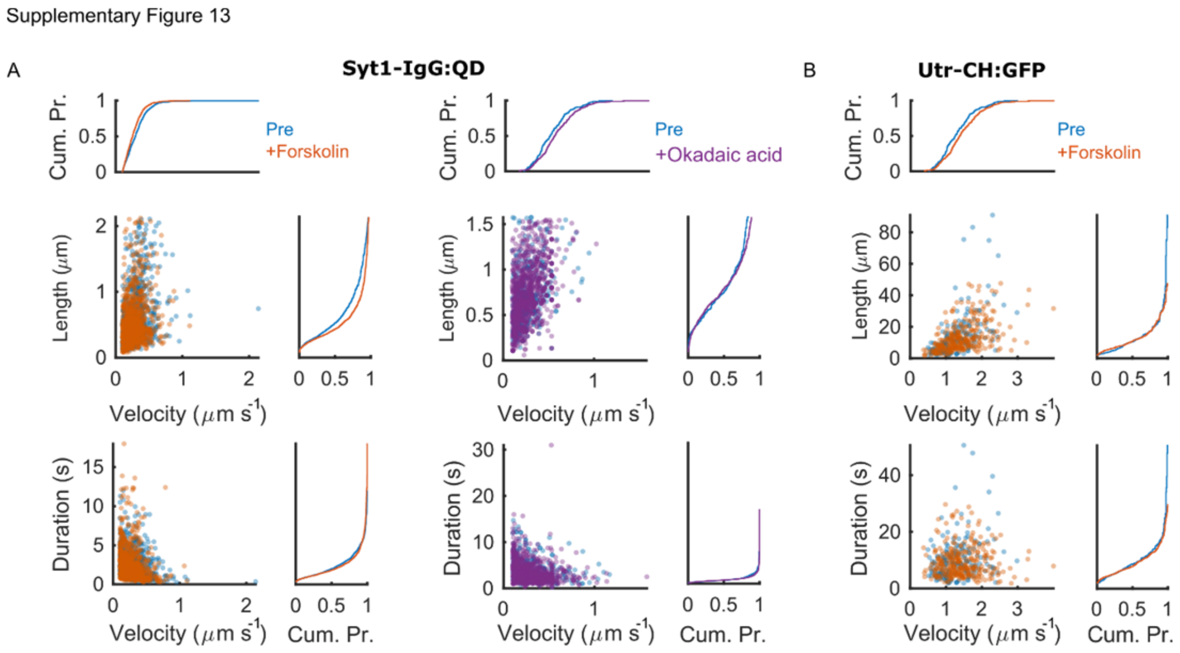

Supplementary Figure 13. Unitary SV AT events and dynamic actin filaments were insensitive to PKA activation

A: In paired imaging experiments, SV traffic was monitored using Syt1-IgG:QDs and the properties of AT transport events were measured before and after treatment with either forskolin (10 μM, 10 min) to activate protein kinase A (PKA) (pre, *N*=681 events, post, *N*=1025 events from 6 coverslips) or okadaic acid to inhibit protein phosphatase 1 and 2a (pre, *N*=421 events, post, *N*=1074 events from 5 coverslips). Unitary events were compared before/after treatment using a mANOVA including unitary velocity, length and duration of AT events and RS tests for post hoc analysis. Forskolin: mANOVA $p<10^{-6}$, median velocity -13.9% ($p<10^{-6}$), median length -18.1% ($p<10^{-6}$), median duration -8.2% ($p>0.07$). Okadaic acid: mANOVA $p=0.48$. B: the same protocol for PKA activation and data analysis was used when actin polymerization was quantified using the probe Utr-CH:GFP. mANOVA $p>0.05$ (pre, *N*=180 filaments; post, *N*=327 filaments from 6 coverslips). Raw data was the same as for main Fig. 5.

Supplementary Figure 14

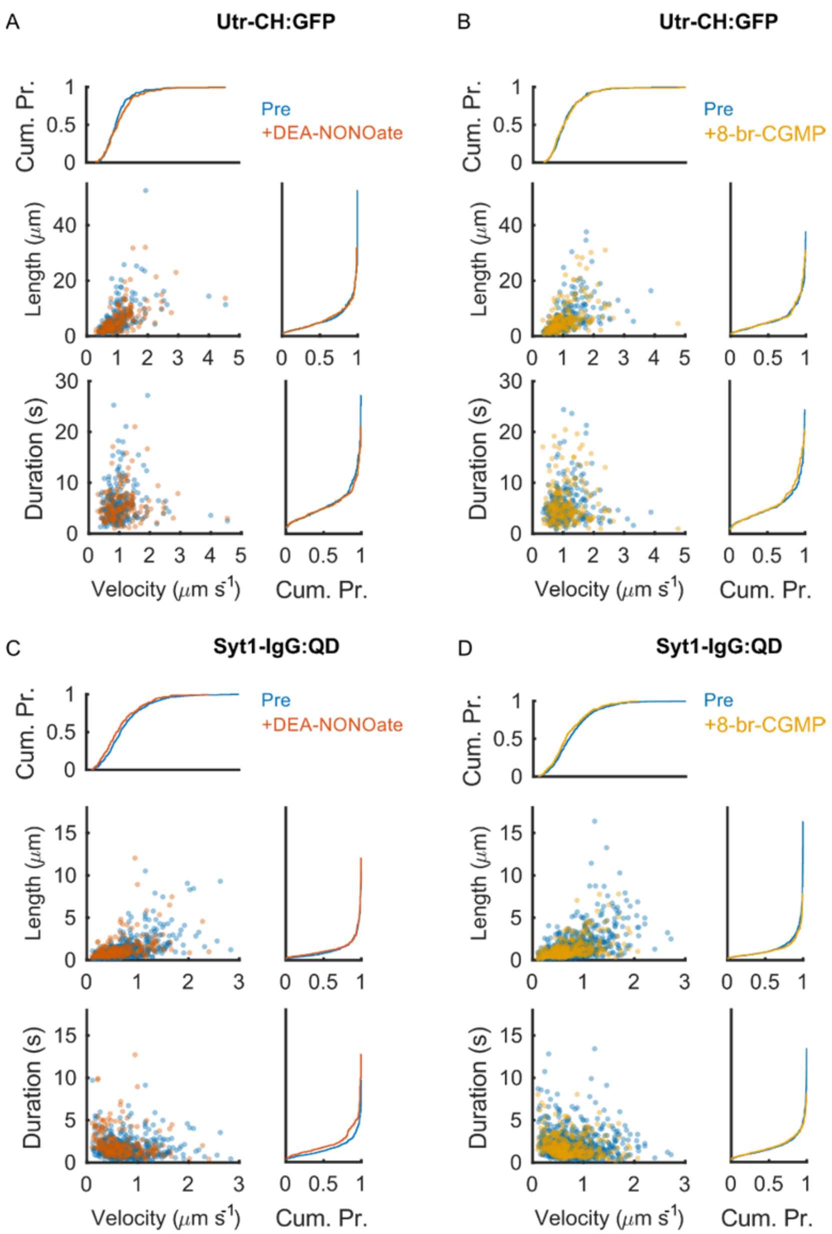

Supplementary Figure 14. Effect of NO on of unitary AT events and unitary growing actin filaments

Unitary properties of actin filaments and SV AT events were measured in paired imaging experiments before and after treatment with DEA-NONOate (3 µM, 4 min), a fast nitric oxide (NO) donor (same raw data as in Fig. 6). Similarly, cGMP concentration was elevated by application of 8-br-cGMP (50 µM, 4 min) to parallel the NO-induced increase in cyclic GMP. A-B: Unitary properties of fast-growing actin filaments as measured in Utr-CH:GFP-expressing

cells before/after treatments. DEA-NONOate: median velocity +1.7% ($p>0.82$, RS test), median length -3.7% ($p>0.45$, RS test), median duration +8.1% ($p>0.1$, RS test) (pre, $N=226$ events from 8 coverslips; post, $N=151$ events). 8-br-cGMP: median velocity +7.0% ($p>0.12$, RS test), median length +15.8% ($p>0.06$, RS test), median duration +5.5% ($p>0.20$, RS test) (pre, $N=267$ events from 7 coverslips; post, $N=192$ events). C-D: SV AT measures before/after treatments. mANOVA for unitary events including velocity, length and duration for the DEA-NONOate treatment: $p<10^{-6}$ (pre, $N=543$ events from 5 coverslips; post, $N=216$ events). Velocity median -11.0% ($p<0.02$, RS test), length median +24.0% ($p<0.005$, RS test), duration median +32.0% ($p<10^{-6}$, RS test). Same analysis for 8-br-cGMP: mANOVA $p=0.084$ (pre, $N=853$ from 4 coverslips; post, $N=329$).

Supplementary Figure 15

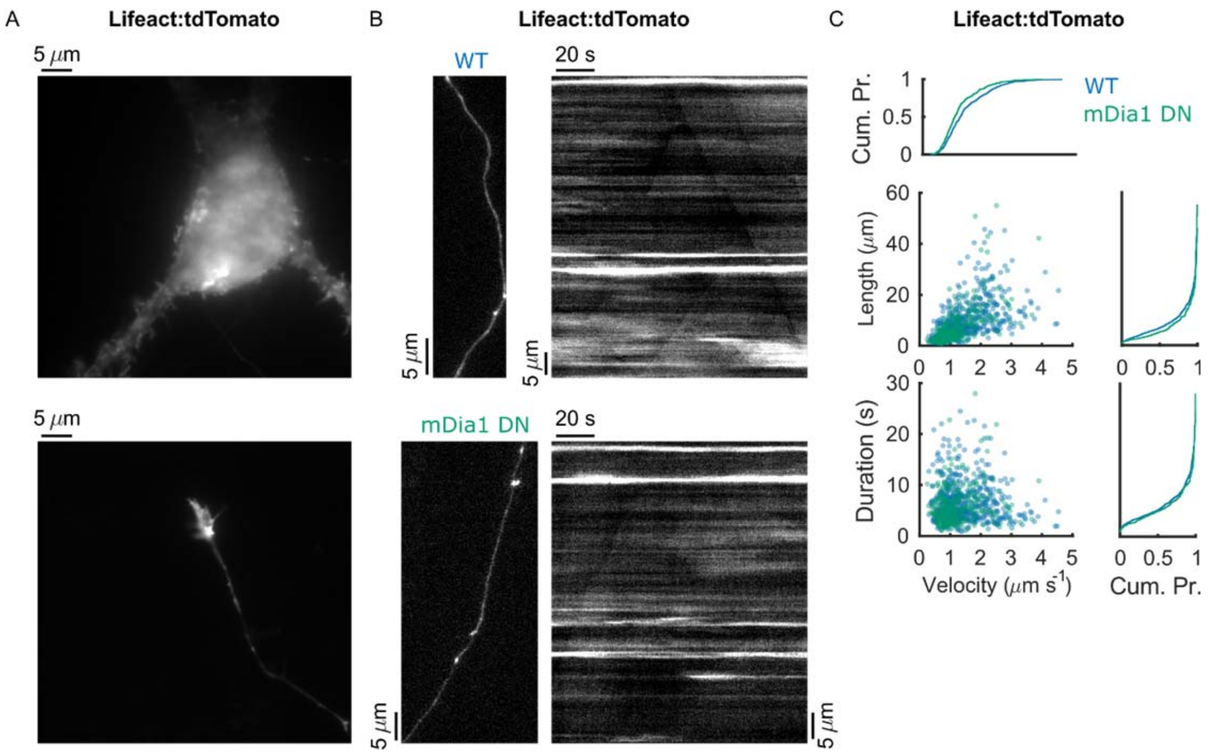

Supplementary Figure 15. Genetic inhibition of mDia1 function impaired the dynamics of actin filaments along axons

A: Somatic (top) and axonal growth cone expression of the genetic probe Lifeact:tdTomato for dynamic F-actin imaging. B: axonal expression of Lifeact:tdTomato (left) and the corresponding fluorescence kymograph for 2 min of 5 Hz time-lapse imaging. Top: fluorescence time-course when Lifeact:tdTomato is expressed alone (WT). Bottom: co-expression with a dominant negative mDia1 construct (mDia1 DN). C: Unitary properties of fast actin filament polymerization measured in WT and mDia1 DN cells. mANOVA WT vs mDia1 DN including length, velocity and duration of filaments: $p<10^{-4}$. Individual properties were moderately changed: velocity median -15.0% ($p<10^{-4}$, RS test), length median -23.0% ($p<10^{-4}$, RS test), duration median -4.0% ($p>0.05$, RS test) (WT N=587, mDia1 DN N=272). Data was from the same coverslips (6 for WT, 8 for mDia1 DN) analyzed in Fig. 7F.

Supplementary Figure 16

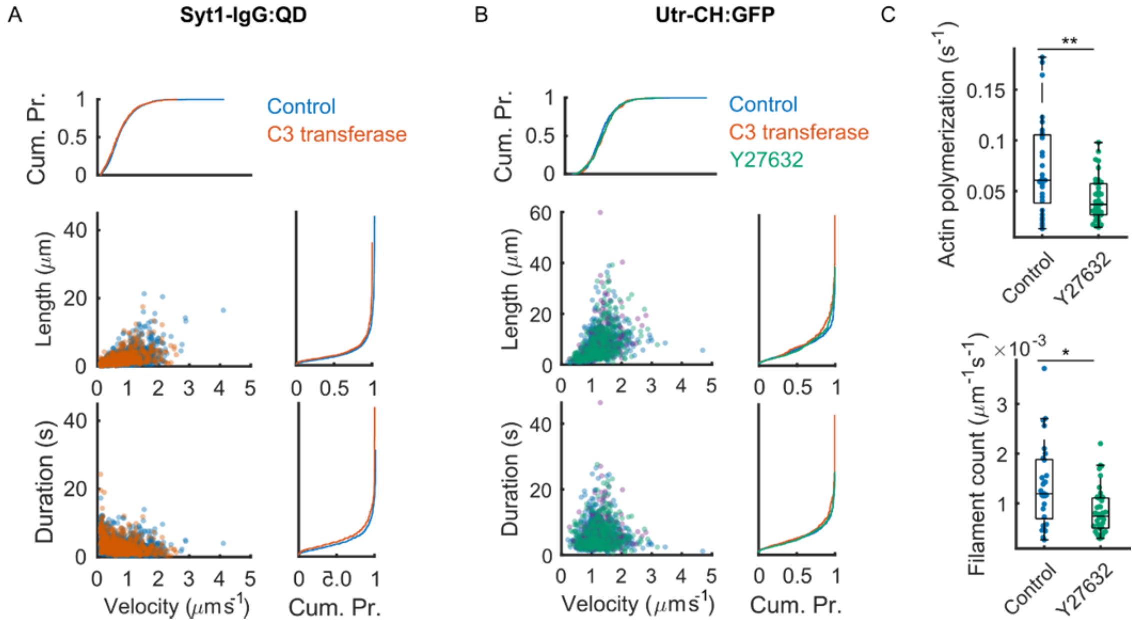

Supplementary Figure 16. Multivariate statistical analysis of unitary AT events and actin filaments after inhibition of RhoA and ROCK

A: Cells were treated with the C3 transferase exoenzyme (2 h, 15 μg ml^{-1}) (*N*=29 movies from 2 coverslips) to inhibit RhoA before being stained with Syt1-IgG:QDs (same raw data as Fig. 6). Unitary SV AT events were then compared to ones observed in untreated coverslips (*N*=29 movies from 3 coverslips). mANOVA with AT unitary length, velocity and duration: $p<10^{-6}$. Median velocity -2.78% (p=0.02, RS test), median length +14.9% ($p<10^{-6}$, RS test), median duration +16.7% ($p<10^{-6}$, RS test) (DMSO, *N*=1898 events; C3, *N*=931 events). B: a similar pharmacological strategy was used when neurons expressed Utr-CH:GFP to characterize the effect of RhoA inhibition on unitary dynamic actin filaments (red). Inhibition of ROCK, a RhoA effector, with Y27632 (2 h, 10 μM) was also investigated (green). 3 groups mANOVA: 1D p= 0.002, 2D p=0.24, indicating that the means of the three groups are contained in a space of a single dimension (DMSO, *N*=530 filaments from 3 coverslips; C3, *N*=209 filaments from 3 coverslips; Y27632, *N*=404 filaments from 4 coverslips). Accordingly, we found: control vs C3 mANOVA: $p<10^{-3}$ and Y27632 vs C3 mANOVA: 1D p=0.23. Overall, it indicated that the treatment with C3 transferase exoenzyme had a significant effect on unitary filament properties when compared to controls, but that it was indistinguishable from that of Y27632. However, the effect of the C3 treatment was non-significant when unitary properties were taken separately: median velocity +7.0% (p>0.11, RS test), median length +12.4% (p>0.13, RS test), median

duration +3.3% (*p*>0.23, RS test). Little changes were also found for the Y27632 treatment: median velocity +5.1% (*p*>0.11, RS test), median length -11.8% (*p*>0.60, RS test), median duration -7.9% (*p*>0.43, RS test). C: Y27632 treatment yielded a large reduction of both the total actin polymerization: median -39.2% (*p*<0.01, RS test) and the frequency of occurrence of new filament polymerization: median -38.0% (*p*<0.02, RS test) (control, *N*=29 movies from 3 coverslips; Y27632, *N*=38 from 4 coverslips). Box plots: median, 25% and 75% percentiles (box) and extreme points (whiskers, excluding outliers).

Supplementary Figure 17

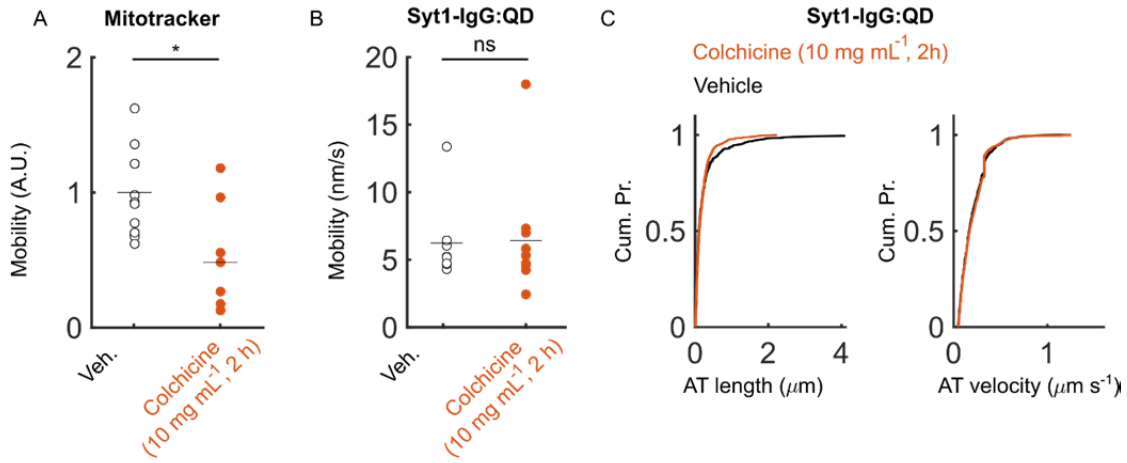

Supplementary Figure 17. Synaptic vesicle traffic is spared by microtubule disruption with colchicine

A: In neurons, colchicine inhibited the traffic of neuronal mitochondria when we measured their total mobility (normalized distance traveled by directed motion). RS test: $p<0.01$ (vehicle, $N=11$ movies; colchicine, $N=8$ movies). B: the mobility of SVs labeled with Syt1-IgG:QDs was spared by colchicine. RS test: $p>0.8$ (veh. $N=8$ movies; colchicine $N=10$ movies). C: Unitary properties of Syt1-IgG:QDs active transport (AT) were compared. AT length: $p>0.1$, RS test. AT velocity: $p>0.5$, RS test (veh., $N=721$ events; colchicine, $N=813$ events).

Supplementary Figure 18

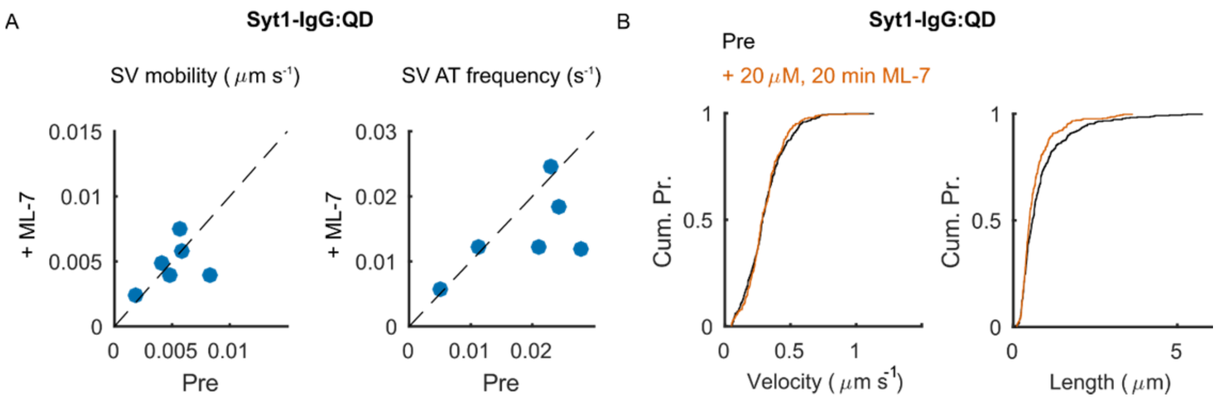

Supplementary Figure 18. SV traffic under myosin light chain kinase inhibition with ML-7

The traffic of SVs labeled with Syt1-IgG:QD was quantified in a paired experiment before and after treatment with 20 μM ML-7 for 15 min. (A) median mobility change was +7.0% (p=1, two-sided sign-test) and median AT frequency change was -9.0% (p=1, two-sided sign-test) (N=6 coverslips). B: Unitary AT velocity was unchanged (median -0.007%, p>0.87%, RS test) while AT length saw a modest change (median -15%, p<0.01, RS test) (pre, N=447 events; post, N=330 events).

Supplementary references

1. Buisson, J., et al., *Intraflagellar transport proteins cycle between the flagellum and its base.* J Cell Sci, 2013. **126**(Pt 1): p. 327-38.
2. de Chaumont, F., et al., *Icy: an open bioimage informatics platform for extended reproducible research.* Nat Methods, 2012. **9**(7): p. 690-6.
3. Beck, A. and M. Teboulle, *Fast gradient-based algorithms for constrained total variation image denoising and deblurring problems.* IEEE Trans Image Process, 2009. **18**(11): p. 2419-34.
4. Olivo-Marin, J.-C., *Extraction of spots in biological images using multiscale products*. Pattern Recognition. Vol. 35. 2002.
5. Chenouard, N., I. Bloch, and J.C. Olivo-Marin, *Multiple hypothesis tracking for cluttered biological image sequences.* IEEE Trans Pattern Anal Mach Intell, 2013. **35**(11): p. 2736-3750.
6. Chenouard, N. and R.W. Tsien. *An algorithm for piecewise-constant velocity estimation and application to particle trajectories in microscopy*. in *2015 IEEE 12th International Symposium on Biomedical Imaging (ISBI)*. 2015.
7. Saxton, M.J. and K. Jacobson, *Single-particle tracking: applications to membrane dynamics.* Annu Rev Biophys Biomol Struct, 1997. **26**: p. 373-99.
8. Henkel, A.W., et al., *Synaptic vesicle movements monitored by fluorescence recovery after photobleaching in nerve terminals stained with FM1-43.* J Neurosci, 1996. **16**(12): p. 3960-7.
9. Harata, N., et al., *Visualizing recycling synaptic vesicles in hippocampal neurons by FM 1-43 photoconversion.* Proc Natl Acad Sci U S A, 2001. **98**(22): p. 12748-53.
10. Darcy, K.J., et al., *Constitutive sharing of recycling synaptic vesicles between presynaptic boutons.* Nat Neurosci, 2006. **9**(3): p. 315-21.
11. Staras, K., et al., *A vesicle superpool spans multiple presynaptic terminals in hippocampal neurons.* Neuron, 2010. **66**(1): p. 37-44.
12. Joensuu, M., et al., *Subdiffractional tracking of internalized molecules reveals heterogeneous motion states of synaptic vesicles.* J Cell Biol, 2016. **215**(2): p. 277-292.
13. Burkel, B.M., G. von Dassow, and W.M. Bement, *Versatile fluorescent probes for actin filaments based on the actin-binding domain of utrophin.* Cell Motil Cytoskeleton, 2007. **64**(11): p. 822-32.
14. Ganguly, A., et al., *A dynamic formin-dependent deep F-actin network in axons.* J Cell Biol, 2015. **210**(3): p. 401-17.
