# Supplementary material for: Synaptic vesicle traffic is supported by transient actin filaments and regulated by PKA and NO
Source: Nat Commun. 2020 Oct 21;11:5318. doi: 10.1038/s41467-020-19120-1 (PMC7578807; doi:10.1038/s41467-020-19120-1)
Supplement: Supplementary file 3 — Reporting Summary [file 41467_2020_19120_MOESM3_ESM.pdf]

nature research

Corresponding author(s): Richard W. Tsien

Last updated by author(s): Aug 27, 2020

Reporting Summary

Nature Research wishes to improve the reproducibility of the work that we publish. This form provides structure for consistency and transparency in reporting. For further information on Nature Research policies, see our Editorial Policies and the Editorial Policy Checklist.

Statistics

For all statistical analyses, confirm that the following items are present in the figure legend, table legend, main text, or Methods section.

n/a	Confirmed	
	x	The exact sample size (n) for each experimental group/condition, given as a discrete number and unit of measurement
	x	A statement on whether measurements were taken from distinct samples or whether the same sample was measured repeatedly
	x	The statistical test(s) used AND whether they are one- or two-sided<br>*Only common tests should be described solely by name; describe more complex techniques in the Methods section.*
x		A description of all covariates tested
	x	A description of any assumptions or corrections, such as tests of normality and adjustment for multiple comparisons
	x	A full description of the statistical parameters including central tendency (e.g. means) or other basic estimates (e.g. regression coefficient) AND variation (e.g. standard deviation) or associated estimates of uncertainty (e.g. confidence intervals)
	x	For null hypothesis testing, the test statistic (e.g. F, t, r) with confidence intervals, effect sizes, degrees of freedom and P value noted<br>*Give P values as exact values whenever suitable.*
x		For Bayesian analysis, information on the choice of priors and Markov chain Monte Carlo settings
x		For hierarchical and complex designs, identification of the appropriate level for tests and full reporting of outcomes
x		Estimates of effect sizes (e.g. Cohen's d, Pearson's r), indicating how they were calculated

Our web collection on statistics for biologists contains articles on many of the points above.

Software and code

Policy information about availability of computer code

Data collection	Andor Solis: image acquisition software program for Andor iXon cameras.<br>NIS-element: image acquisition software program for Nikon confocal microscopes.
Data analysis	Most basic image analysis tasks were performed using the ICY open-source platform (http://icy.bioimageanalysis.org/).<br>Kymograph extraction: 'KymographTracker' plugin (ID: ICY-K4O2C2)<br>Spot detection: "Spot detector" plugin (ID: ICY-R3M2Y2)<br>Vesicle tracking: "Spot Tracking" plugin (ID: ICY-L5S9M5)<br>All other analysis scripts, including Monte Carlo simulations, were produced using Matlab. Major scripts will be made available at the time of publication upon reasonable request.

For manuscripts utilizing custom algorithms or software that are central to the research but not yet described in published literature, software must be made available to editors and reviewers. We strongly encourage code deposition in a community repository (e.g. GitHub). See the Nature Research guidelines for submitting code & software for further information.

Data

Policy information about availability of data

All manuscripts must include a data availability statement. This statement should provide the following information, where applicable:

- Accession codes, unique identifiers, or web links for publicly available datasets
- A list of figures that have associated raw data
- A description of any restrictions on data availability

All data analyzed in this study are included in the manuscript and in the supplementary information file.
Large, raw image data sets will be made available upon reasonable request.

Field-specific reporting

Please select the one below that is the best fit for your research. If you are not sure, read the appropriate sections before making your selection.

[x] Life sciences [ ] Behavioural & social sciences [ ] Ecological, evolutionary & environmental sciences

For a reference copy of the document with all sections, see nature.com/documents/nr-reporting-summary-flat.pdf

Life sciences study design

All studies must disclose on these points even when the disclosure is negative.

Sample size	Statistical methods to estimate sample size were not systematically used. For paired experiments (before/after treatment) the minimal sample size was straightforwardly determined by assuming a priori a reliable drug effect.
Data exclusions	No data was excluded from the analysis.
Replication	Repetitions of each experiment were spread over multiple weeks to minimize the influence of each day setup. An equal number of test and control experiments were performed each day.
Randomization	On each experimental day, coverslips covered with neurons were taken and randomly assigned to a test drug or control (eg DMSO) before starting the experiment and examining the cells on the microscope. The number of coverslips in each group was balanced daily.
Blinding	Most analysis methods were automated and were applied in a, de facto, blind manner. Specific blinding was used for semi-automated processing (trajectory tracing in kymographs) so that the operator could not know the groups when analyzing the data.

Reporting for specific materials, systems and methods

We require information from authors about some types of materials, experimental systems and methods used in many studies. Here, indicate whether each material, system or method listed is relevant to your study. If you are not sure if a list item applies to your research, read the appropriate section before selecting a response.

Materials & experimental systems

n/a	Involved in the study	
[ ]	[x]	Antibodies
[x]	[ ]	Eukaryotic cell lines
[x]	[ ]	Palaeontology and archaeology
[ ]	[x]	Animals and other organisms
[x]	[ ]	Human research participants
[x]	[ ]	Clinical data
[x]	[ ]	Dual use research of concern

Methods

n/a	Involved in the study	
[x]	[ ]	ChIP-seq
[x]	[ ]	Flow cytometry
[x]	[ ]	MRI-based neuroimaging

Antibodies

Antibodies used	Synaptic Systems Mouse monoclonal anti synapsin 1 (105 311 BT). Biotinylated.<br>Synaptic Systems Mouse monoclonal anti synapsin 1 (105 311 CR1). Chromeo 488-conjugated.<br>Sigma-Aldrich Mouse monoclonal anti alpha-tubulin (T6074).
Validation	Synaptic Systems anti synapsin 1 antibodies were tested in rats: Qin X, Tsien RW, Park H, Biochemical and biophysical research communications (2019). The colocalization with FM-dye staining of recycling synaptic vesicles was checked in Supplementary Figures.<br>Monoclonal anti alpha-tubulin recognizes an epitope located at the C-terminal end of alpha-tubulin isoform in a variety of organisms (eg, human, mouse, bovine, rat, African green monkey, kangaroo rat, chicken, sea urchin, and chlamydomonas) (from Sigma-Aldrich).

	Experimental valication in M. LeDizet and G. Piperno, Methods in Enzymology, vol 196, 1991.

Animals and other organisms

Policy information about studies involving animals; ARRIVE guidelines recommended for reporting animal research

Laboratory animals	Sprague-Dawley rat pups (1-6 hours after birth) born on site. Impregnated animals were purchased from Charles River.
Wild animals	*Provide details on animals observed in or captured in the field; report species, sex and age where possible. Describe how animals were caught and transported and what happened to captive animals after the study (if killed, explain why and describe method; if released, say where and when) OR state that the study did not involve wild animals.*
Field-collected samples	*For laboratory work with field-collected samples, describe all relevant parameters such as housing, maintenance, temperature, photoperiod and end-of-experiment protocol OR state that the study did not involve samples collected from the field.*
Ethics oversight	All procedures involving animals were approved by the Institutional Animal Care and Use Committee at the New York University Langone Medical Center (NYULMC), and in accordance with guidelines from the National Institutes of Health.

Note that full information on the approval of the study protocol must also be provided in the manuscript.
